# Fluoride Regulate Osteoblastic Transforming Growth Factor-β1 Signaling by Mediating Recycling of the Type I Receptor ALK5

**DOI:** 10.1371/journal.pone.0170674

**Published:** 2017-01-26

**Authors:** Chen Yang, Yan Wang, Hui Xu

**Affiliations:** School of Pharmaceutical Sciences, Jilin University, Changchun, P. R. China; State University of New York, UNITED STATES

## Abstract

This study aimed to preliminary investigate the role of activin receptor-like kinase (ALK) 5 as one of TGF-βR1 subtypes in bone turnover and osteoblastic differentiation induced by fluoride. We analyzed bone mineral density and the expression of genes related with transforming growth factor-β1(TGF-β1) signaling and bone turnover in rats treated by different concentrations of fluoride with or without SB431542 *in vivo*. Moreover, MTT assay, alkaline phosphatase staining, RT-PCR, immunocytochemical analysis and western blot analysis were used to detect the influence on bone marrow stem cells (BMSC) after stimulating by varying concentration of fluoride with or without SB431542 *in vitro*. The *in vivo* study showed SB431542 treatment affected bone density and gene expression of rats, which indicated TGF-β1 and ALK5 might take part in fluoride-induced bone turnover and bone formation. The *in vitro* study showed low concentration of fluoride improved BMSC cells viability, alkaline phosphatase activity, and osteocalcin protein expression which were inhibited by high concentration of fluoride. The gene expression of Runx2 and ALK5 in cells increased after low concentration fluoride treatment which was also inhibited by high concentration of fluoride. Fluoride treatment inhibited gene and protein expression of Samd3 (except 1 mgF^-^/L). Compared with fluoride treatment alone, cells differentiation was inhibited with SB431542 treatment. Moreover, the expression of Runx2, ALK5 and Smad3 were influenced by SB431542 treatment. In conclusion, this preliminary study indicated that fluoride regulated osteoblastic TGFβ1 signaling in bone turnover and cells differentiation via ALK5.

## Introduction

Fluoride is an important element for human in maintaining bone strength and stimulates bone growth [1]. Moreover, it regulates bone formation by enhancing osteoblast differentiation and stimulating alkaline phosphatase (ALP) activity, which considered a marker for osteoblast [2,3]. However, excessive fluoride may result in skeletal fluorosis, a condition that patients display various bone lesions including osteosclerosis, osteoporosis and degenerative joint changes [4,5]. Although some investigators have reported many studies in fluoride and bone turnover [6,7], the pathogenic mechanism of the skeletal fluorosis was still unclear.

Previous study concluded that fluoride exerted influence on bone turnover by regulating certain factors such as runt-related transcription factor 2 (Runx2) and receptor activator for nuclear factor-κ B ligand (RANKL), which was considered as key factors for osteoblast and osteoclast differentiation. Besides, transforming growth factor-β1 (TGF-β1) is known to be essential for osteoblast and osteoclast differentiation [8]. ALK5 is a key factor of TGF-β1 signaling, inhibition of the binding of ALKS to substrate Smad2/Smad3 or phosphorylating substrate Smad2/Smad3 leads to the blockage of the transduction of TGF-β1 signal. Once activated, these Smads proteins in combination with Smad4 would regulate the transcription of target genes [9,10]. Moreover, previous studies found seven activin-receptor like kinases (ALKs)1-7 of type I receptors and five type II receptors for TGF-β1 signaling transferred [11].

Numerous studies concluded that many factors affected TGF-β1 signaling pathway [12,13]. Suzuki *et al* [14] concluded that fluoride down-regulated TGF-β1 signaling and attenuated kallikrein related peptidase (KLK)4 expression in fluorosed enamel. Therefore, we supposed that TGF-β1 signaling might have correlation with fluoride on osteoblastic differentiation. In addition, SB431542 is a selective TGF-β1 inhibitor, it inhibits the activity of ALK5 (TGF-β type I receptor) [15]. TheSmad2/3 proteins are substrates for ALK5. Therefore, SB431542 was used in this study to investigate the mechanism of fluoride induced osteoblast differentiation via ALK5 pathway.

## Methods

### Animals and treatment

The male Wistar rats (6 weeks old, 150g) used in the study were provided by the Experimental Animal Center of Bethune Medical College, Jilin University. The study protocol was subject to approval by the Ethics Committee on the Use and Care of Animals of Jilin University (Changchun, China). All experimental animals implemented anesthesia before they were euthanized by cervical dislocation. Each rat was kept in an individual cage with a standard environment. The rats were randomly divided into control group, low fluoride group and high fluoride group (n = 20 for each group). One third of rats were treated with sodium fluoride (NaF, Sigma–Aldrich Co., USA) by gavage at a dose of 10 mg fluoride/kg.bw as low fluoride, and one third of rats were treated with 20 mg fluoride/kg.bw as high fluoride and the remaining were kept as the control group. The doses of fluoride were selected based on past reports [16]. After one month, half of rats in each group were injected with an ALK5 inhibitor (SB431542; Selleck Chemicals Co., USA) at a dose of 2.1 mg/kg.bw as previously described [17]. Rats were divided into six groups which were designated as control, control + SB431542, 10 mg F^-^/kg.bw,10 mg F^-^/kg.bw + SB431542, 20 mg F^-^/kg.bw and20 mg F^-^/kg.bw + SB431542. After four weeks of SB431542 treatment, bone mineral density of the rats in each group was measured by a Discovery WA Scanner (Hologic; Marlborough, MA, USA) with positioning the legs of rats on a platform.

### Reverse transcription and real-time PCR of bone tissue

After treatment, a sample of fresh bone tissue was obtained from each rat. Bone was immersed and grinded in liquid nitrogen, then total RNA was extracted by TRIzol reagent (Invitrogen; Carlsbad, CA, USA) and quantified by scanning spectrophotomer. Next, cDNA was prepared by using a Transcriptor First Strand cDNA Synthesis Kit (Applied Biosystems, USA), and genes expression of the bone samples was examined by real-time PCR using SYBR green detection method (Applied Biosystems, USA). All the primers were validated before PCR reaction. Fold change was calculated using ddCT method (2^∧^-ddCt). Glyceraldehyde-3-phosphate dehydrogenase (GAPDH) was used as a reference. All experiments were repeated three times. The primers used in the study were synthesized by Sangon Biotech (Shanghai, China) and were shown in Table 1.

**Table 1:** Primers used for real-time PCR for rats.

Genens	Number	Sequences(5′-3′)
Runx2	NM_053470	Forward:CGCATTCCTCATCCCAGTAT Reverse:GCCTGGGGTCTGTAATCTGA
RANKL	NM_057149.1	Forward:TCAGGAGTTCCAGCTATGAT Reverse:CCATCAGCTGAAGATAGTCC
Smad3	NM_013095.3	Forward:GGCAGGATGTTTCCAGCTA Reverse:GCCAGTCCACAGACCATGTCA
ALK5	NM_012775.2	Forward:ACCTTCTGATCCATCCGTT Reverse:CGCAAAGCTCAGCCTAG
GAPDH	M17701.1	Forward:TGATTCTACCCACGGCAAGTT Reverse:TGATGGGTTTCCCATTGATGA

### Bone marrow stem cells culture and treatment

Bone marrow stem cells (BMSC) were isolated from the femurs of young Kunming mice (3 weeks old, 30g) in a sterile environment. Cells were washed out by phosphate-buffered saline (PBS, pH = 7.4) from marrow cavity at 4°C, then, they were collected and centrifuged for 10 min at 1800rpm. After that, cells were cultured in DMEM/F12 medium (Hyclone Co, USA) containing mineralization induction agents (0.05 g/L vitamin C, 40 ng/mL dexamethasone and 10 mmol/L β-sodium glycerol phosphate) and 10% fetal bovine serum at 37°C with 5% CO_2_. After two weeks of induced culturing, according to our previous study [18], cells were seeded into a 96-well plate at a density of 1×10^4^ cells per well, and then were treated with fluoride at concentrations of 1 mg/L, 4 mg/L and 16 mg/L with or without 10 μmol/L SB431542 as previously described [19]. Control group was cultured in medium without fluoride and SB431542.

### MTT assay

MTT assay was performed to detect cells viability at 4-day and 7-dayafter exposure to fluoride and SB431542. Cells were treated with MTT reagent (5mg/ml), then incubated for 4 h at 37°C with 5% CO_2_. Following incubation, dimethyl sulfoxide (DMSO, Sigma-Aldrich, USA) was added to allow color development, and the optical density (OD) of each well at 490nm was measured with a spectrophotometer. Cell viability was calculated as follows:

cell viability = [OD (experimental) - OD (blank)] / [OD (control) - OD (blank)].

### Alkaline phosphatase staining

Cells were cultured by fluoride at concentrations of 1 mg/L, 4 mg/L and 16 mg/L with or without 10 μmol/L SB431542 and 10 μg/L TGF-β1 for 7 days as previously described [20]. Next, cells were washed by PBS and fixed with 90% alcohol for 10 min at 4°C, then, they were treated with an incubation solution (2% sodium pentobarbital, 25 mL, 2% β-sodium glycerophosphate, 25 mL, 2% magnesium chloride, 2 mL, 2% calcium nitrate, 8 mL, and acetone, 40 mL) at 37°C for 4 h. Then, cells were stained with 2% cobalt nitrate solution and subsequently with 1% ammonium sulfide. After staining, cells were photographed, and ALP-positive cells displayed a dark-brown color.

### Reverse transcription and real-time PCR of BMSC

Cells were exposed to fluoride at concentrations of 1 mg/L, 4 mg/L and 16 mg/L with or without 10μmol/L SB431542 for 7 days. Following treatment, total RNA was extracted by using TRIzol reagent (Invitrogen; Carlsbad, CA, USA) and quantified by a scanning spectrophotometer. First-strand cDNA was synthesized from 1 μg of total RNA using an Oligo(dT) 18 primers and reverse transcriptase (Applied Biosystems, USA). The real-time PCR were performed under the same procedures stated above. All experiments were repeated three times. The primers used were synthesized by Sangon Biotech (Shanghai, China) and were shown in Table 2.

**Table 2:** Primers used for real-time PCR for mice.

Genens	Number	Sequences(5′-3′)
Runx2	NM_001278484.2	Forward:CCGGTCTCCTTCCAGGAT Reverse:GGGAATCTGCTGTGGCTTC
Smad3	NM_016769.4	Forward:CTGGGCCTACTGTCCAATGT Reverse:CATCTGGGTGAGGACCTTGT
ALK5	NM_001312869.1	Forward:GGAAATTGCTCGACGCTGTT Reverse:TTCTCATTTCTTCAACCGATGGA
GAPDH	XM_011241214.1	Forward:GGCGCCCAGAACATCAT Reverse:CGGACACATTGGGGGTAG

### BMSC immunocytochemical analysis

The immunocytochemical analysis was utilized to detect the expression of osteocalcin (OCN) in BMSC. Cells were exposed to different concentrations of fluoride with or without 10 μmol/L SB431542 for 28 days. At the end of incubation, the cells were washed in PBS, fixed with 4% paraformaldehyde for 20 min at 4°C, and treated with 0.5% TritonX-100 for 15 min. Next, they were blocked with 10% H_2_O_2_ at room temperature for 10 min, then treated with 3% bovine serum albumin (BSA) at room temperature for 30 min. The cells were then incubated overnight at 4°C with OCN rabbit polyclonal antibody (OCN, 1:100; Santa Cruz, USA) which was diluted in 2% BSA/0.1M PBS, and cells were further incubated with a goat anti-rabbit IgG at room temperature for 1h. The cell coverslips sections were treated with an avidin-biotin-peroxidase (Maixin Biotech Co, China) complex for 30min. Then, they were immersed in diaminobenzidine (Maixin Biotech Co, China) for 1 min. Hematoxylin was used for nuclear counterstaining.

### Western blot analysis for Smad2/3 and P-Smad2/3

Smad2/3 and phosphorylation of Smad2/3 expression were measured by western bolt analysis. Cells were lysed with the lysis buffer (Beyotime, China), and the protein concentration was quantified using the bicinchoninic acid (BCA) method. Samples of protein (20 μg) were separated on 10% SDS-PAGE gels, and transferred onto PVDF membranes (PALL, USA). The transfer membranes were then blocked with 5% milk-TBST (20 mmol/L Tris-HCl (pH 8.0), 8 g/L NaCl, and 0.1% Tween 20) for 1h at room temperature. After blocking, the membranes were incubated with primary antibodies (GAPDH, 1:5000; Smad2/3, 1:800; P-Smad2/3, 1:500; Santa Cruz Co, USA) at 4°C overnight, then incubated with a secondary antibody (goat anti-rabbit IgG-HRP, 1:3000; Santa Cruz Co, USA) for 2h. Finally, immunoreactive bands were detected by EasySee Western Blot Kit (Transgen, China). The staining results were analyzed by Gel-Pro Analyzer 4.0 software. The expression levels of individual proteins were indicated as a ratio relative to GAPDH expression.

### Statistical analysis

All data were expressed as the mean ± SD. Statistical differences between the groups were analyzed using LSD and Duncan’s test, Statistical analysis was performed by SPSS 13.0 software (SPSS Inc, Chicago). A P-value of less than 0.05was considered statistically significant.

## Results

### Fluoride influenced bone mineral density in rats

**Fig 1:**
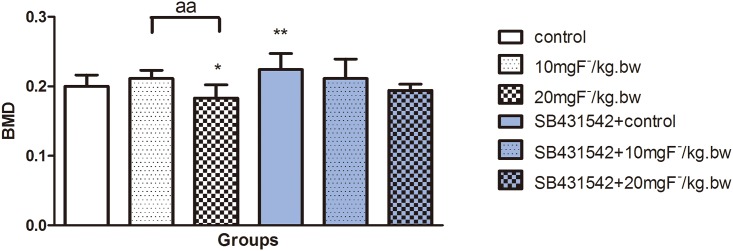
Changes of bone mineral density in rats treated by fluoride with or without SB431542. Rats was treated with sodium fluoride by gavage at 10 mgF^-^/kg.bw and 20 mgF^-^/kg.bw for 2 months, and half of rats in each group were injected with an ALK5 inhibitor (SB431542, 2.1 mg/kg.bw). The tibias were collected and bone mineral density was measured by using a Hologic Discovery WA scanner. Results are expressed as mean±SD (n = 10). Results indicated significant changes (*P < 0.05, compare with control group; aa P < 0.01, compare with two groups).

As an indicator of bone mass bone mineral density (BMD) implies the bone turnover state [21]. Our results showed BMD decreased in fluoride-treated rats and it was significantly reduced in high dose of fluoride group compared to the control and low dose of fluoride groups. Moreover, SB431542 treatment enhanced BMD in rats, and showed a better BMD result compared to the fluoride treatment alone (Fig 1). The results indicated that SB431542 might influence the change of bone mass induced by fluoride.

### Fluoride regulated gene expression in bone tissue of rats

**Fig 2:**
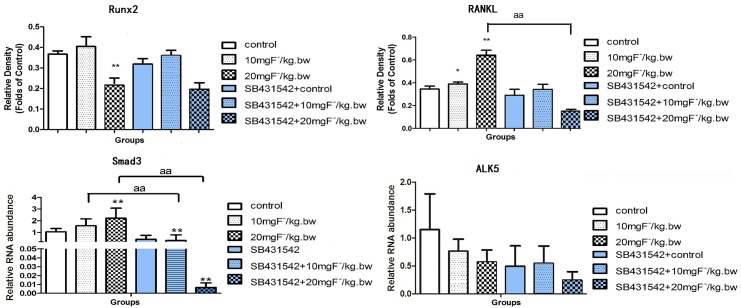
Gene expression of Runx2, RANKL, Smad3 and ALK5 in rats treated by fluoride with or without SB431542. Rats was treated with sodium fluoride by gavage at 10 mgF^-^/kg.bw and 20 mgF^-^/kg.bw for 2 months, and half of rats in each group were injected with an ALK5 inhibitor (SB431542, 2.1 mg/kg.bw). The femurs were collected and extracted mRNA by Trizol reagent. Realtime PCR was used to analyze Runx2, RANKL, Smad3 and ALK5 expression. Results are expressed as mean± SD(n = 3). (**P < 0.01 compare with control group; aa P < 0.01, compare with two groups).

Gene expression ofRunx2, RANKL, ALK5 and Smad3 was detected in bone tissue. Results showed that gene expression ofRunx2 slightly enhanced after low-dose fluoride treatment, but decreased in high-dose fluoride group compared with control group. Moreover, fluoride treatment increased RANKL expression and significantly higher expression was observed in high-dose fluoride treatment group. The expressions of Runx2 and RANKL were inhibited in co-treatment with fluoride and SB431542 compared to the fluoride treatment alone. The gene expression of intracellular signaling factors of TGF-β1 signaling, such as ALK5 and Smad3, were also measured. Results indicated that fluoride treatment stimulated expression of Smad3 compared to the control, but SB431542 administration with fluoride markedly inhibited Smad3comparedwith fluoride treatment alone. The expression of ALK5 was slightly lower than that in the control group, and SB431542 co-treated with fluoride further decreased ALK5 expression, which might attribute to the activity decreases of osteoblastic cells mentioned in above results(Fig 2). These results indicated that TGF-β1 might regulate fluoride induced bone turnover by mediating Smad3.

### Fluoride and SB431542 treatment influenced BMSC viability

**Fig 3:**
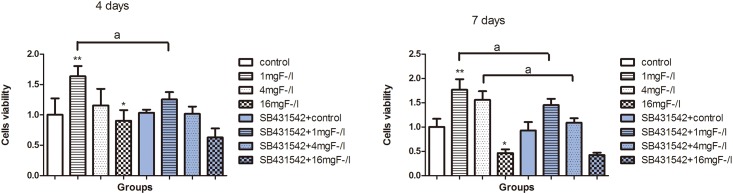
Cell viability of BMSC exposed to fluoride with and without SB431542. Cells were treated with 1 mg/L, 4 mg/L, and 16 mg/L of fluoride with or without 10μmol/L SB431542 for 4 and 7 days. MTT assay was used to detect cell viability. Absorbance was measured at 490nm in aspectrophotometer. Average optical density (OD) value of cells viability was represented as mean±SD (n = 8) (*P < 0.05, **P < 0.01 compare with control group; aa P< 0.05, compare with SB431542 group).

Results showed that 1, 4 mg/L of fluoride treatment enhanced the viability of BMSC at 4-day and7-day, while the viability significantly decreased in cells exposed to16 mg/L of fluoride compared with the control. SB431542 co-treated with 1 mg/L fluoride inhibited cells viability compared with fluoride treatment alone after 4 days of culture. SB431542 co-treated with 1 mg/L and 4 mg/L fluoride inhibited cells viability compared with fluoride treatment alone after 7 days of culture (Fig 3). These results indicated that low dose of fluoride increased cell viability, while high dose inhibited it. Co-exposure to SB431542and fluoride significantly influenced cells viability.

### Fluoride regulated the ALP activity in BMSC

**Fig 4:**
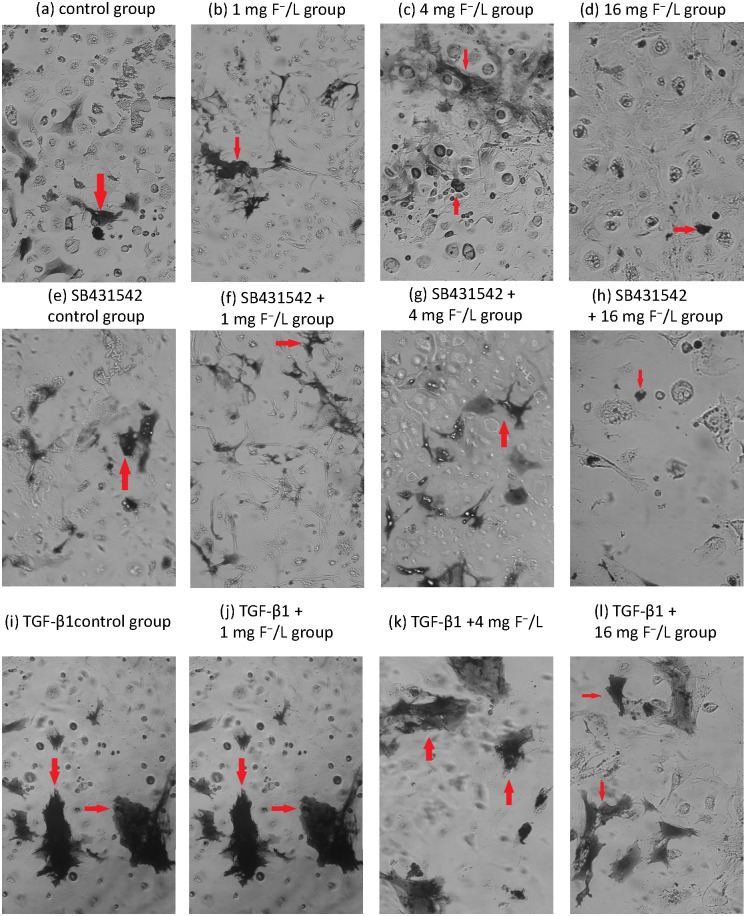
Activity of Alkaline phosphatase of cells exposed to fluoride with and without SB431542. Cells were treated with 1 mg/L, 4 mg/L, and 16 mg/L of fluoride with or without 10μmol/L SB431542 for 7 days. The deposition of Cobalt sulfide particles was stained into light black granules in cytoplasm. The positive staining for deposition of Cobalt sulfide particles was shown in control group (a), 1 mg F^−^/L group (b), 4 mg F^−^/L (c), 16 mg F^−^/L group (d), SB431542 control group (e), SB431542 +1 mg F^−^/L group (f), SB431542 +4 mg F^−^/L (g), SB431542 + 16 mg F^−^/L group (h), TGF-β1control group (i), TGF-β1 + 1 mg F^−^/L group (j), TGF-β1 +4 mg F^−^/L (k), TGF-β1 + 16 mg F^−^/L group (l) under microscopy (red arrowheads; scale bar, 50 μm).

The ALP regulated calcium deposition and was considered as a key factor in osteoblast early differentiation process. Results showed that treatment with fluoride at concentrations of 1 mg/L and 4 mg/L increased the levels of ALP positive staining in cells, while dark staining area was reduced after 16 mg/L fluoride treatment compared with control (Fig 4a–4d). Cells treated with SB431542 and 1 mg/L fluoride showed less ALP positive staining compared with fluoride treatment alone (Fig 4f). Cells treated with TGF-β1 showed higher levels of ALP-positive staining compared with control (Fig 4i). Furthermore, ALP positive staining was increased after fluoride combined with TGF-β1 treatment compared with fluoride treatment alone (Fig 4i–4l). These results showed that TGF-β1 significantly enhanced ALP activity in cells induced by fluoride.

### Fluoride regulated gene expression of BMSC

**Fig 5:**
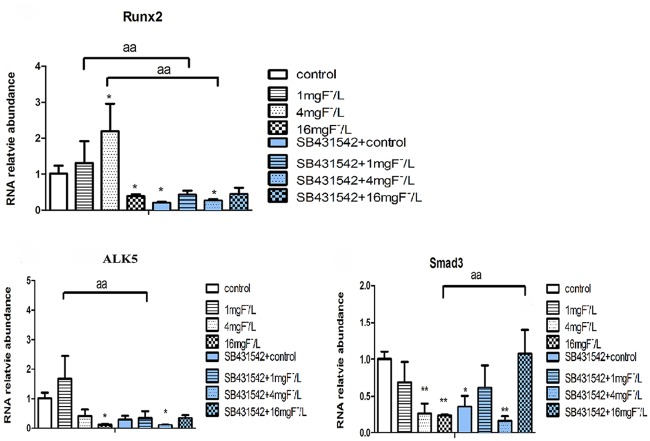
Expression of Runx2, Smad3 and ALK5 in cells BMSC exposed to fluoride with and without SB431542. The BMSCs were collected and extracted mRNA by Trizol reagent. Realtime PCR was used to analyze Runx2, Smad3 and ALK5 expression. The GAPDH was used as inner control. Results are expressed as mean± SD (n = 3). (*P < 0.05, **P < 0.01 compare with control group; aa P < 0.01, compare with two groups).

We detected the expression of Runx2, ALK5 and Smad3 in BMSCs. Results showed that compared with control, Runx2 gene expression increased by stimulation with 1 mg/L and 4 mg/L fluoride, but it was inhibited by 16 mg/L fluoride treatment. The level of ALK5 gene expression increased by 1 mg/L fluoride treatment while it was inhibited by 16 mg/L fluoride treatment compared with control. The expression of Smad3 markedly decreased in cells treated with 4 mg/L and 16 mg/L of fluoride compared with control. With SB431542 treatment in cells, Runx2 expression significantly decreased compared with fluoride treatment alone. The ALK5 expression was inhibited by 1 mg/L fluoride and SB431542 co-treatment compared with fluoride treatment alone. While the expression of Smad3 increased with 16 mg/L fluoride and SB431542 co-treatment (Fig 5).

### OCN protein expression in BMSC treated with fluoride and SB431542

**Fig 6:**
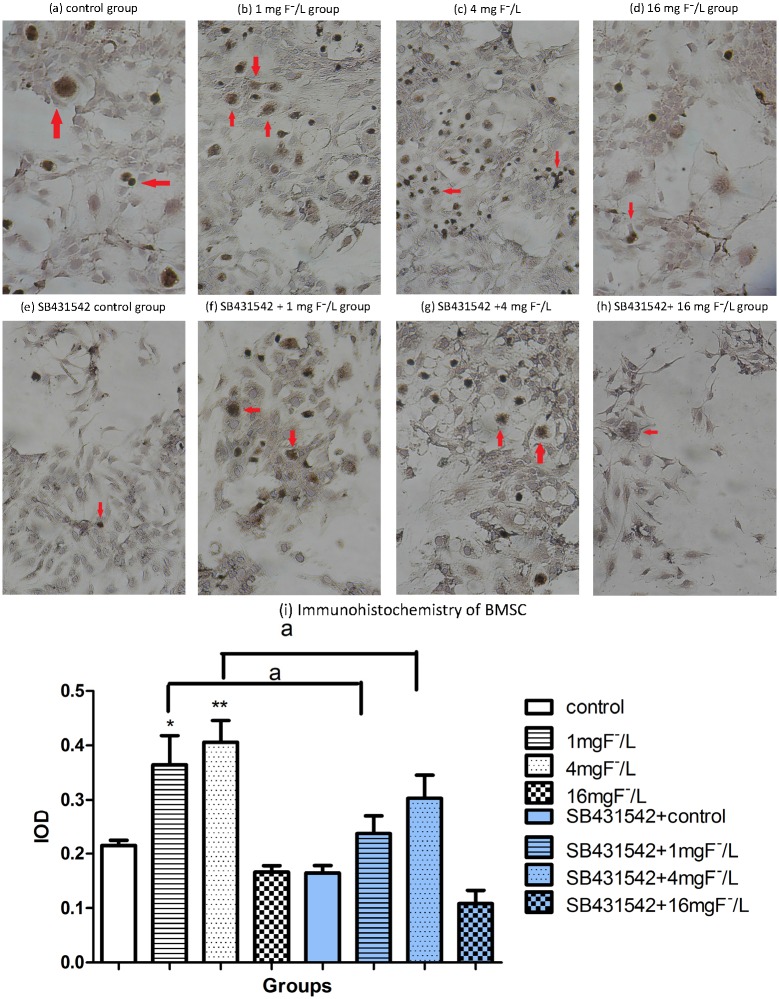
Protein level of osteocalcin in BMSC exposed to fluoride with and without SB431542. Cells were treated with 1 mg/L, 4 mg/L, and 16 mg/L of fluoride with or without 10μmol/L SB431542 for 28 days. Immunocytochemistry analysis was used to test osteocalcin expression in situ. Positive staining showed brown granules in cytoplasm as that in control group (a), 1 mg F^−^/L group (b), 4 mg F^−^/L (c),16 mg F^−^/L group (d), SB431542 control group (e), SB431542 + 1 mg F^−^/L group (f), SB431542 +4 mg F^−^/L (g), SB431542+ 16 mg F^−^/L group (h) under microscopy (red arrowheads; scale bar, 50 μm). Immunocytochemistry was analyzed by using Image-Pro Plus 6.0 software to measure integrated optical density. Results are expressed as mean±SD (n = 3). Results indicated the significant changes(*P < 0.05, **P < 0.01 compare with control group; a P< 0.05, compare with two groups).

The OCN is commonly used as a marker for osteoblast differentiation. As it was shown in Fig 6, OCN protein was highly expressed in BMSC cells treated with 1 mg/L and 4 mg/L concentrations of fluoride, while OCN staining was slightly reduced by 16 mg/L fluoride treatment compared to control. SB4314542 co-treated with 1 mg/L and 4 mg/L fluoride significantly inhibited OCN protein expression compared with fluoride alone. These results indicated that OCN protein expression was inhibited by excessive fluoride and SB431542 treatment.

### Protein expression of Smad2/3 and p-Smad2/3 in BMSC treated with fluoride and SB431542

**Fig 7:**
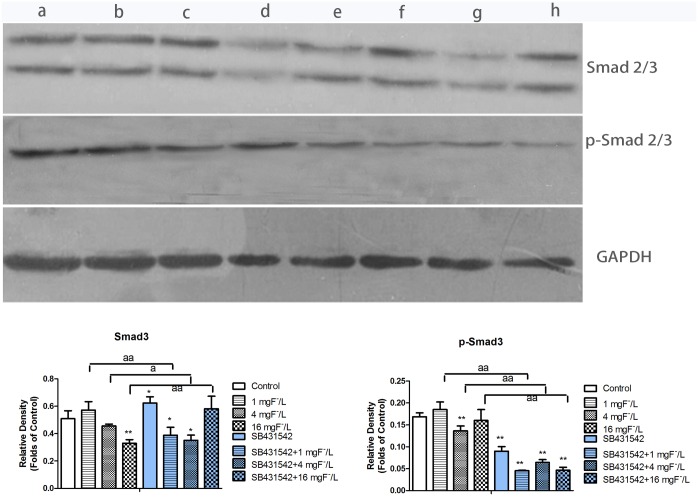
Protein level of phosphorylation of Smad2/3 in BMSC exposed to fluoride with and without SB431542. Cells were lysed with lysis buffer. Proteins were separated by SDS-PAGE and transferred to PVDF membrane, which were incubated with primary and secondary antibodies. Immunostained proteins were detected by EasySee Western Blot Kit. Result was shown in control group (a), 1 mg F−/L group (b), 4 mg F−/L (c), 16 mg F−/L group (d), SB431542 control group (e), SB431542 + 1 mg F^−^/L group (f), SB431542 +4 mg F^−^/L (g), SB431542+ 16 mg F^−^/L group (h). The gels were analyzed by using Gel-Pro Analyzer 4.0 software to measure integrated optical density. Results are expressed as mean±SD (n = 3). Results indicated the significant changes (*P < 0.05, **P < 0.01 Vs control group; a P< 0.05, compare with two groups, aa P< 0.01, compare with two groups).

P-Smad2/3 protein is an important downstream mediator of TGF-β1/ALK5 signaling in regulating osteoblast differentiation. We measured p-Smad2/3 and Smad2/3 expressions in BMSCs treated by fluoride and ALK5 inhibitor (SB431542). As shown in Fig 7, the expression of p-Smad2/3 slightly increasedafter1mg/L of fluoride treatment compared with control, which was consistent with protein level of Smad2/3, but other concentrations of fluoride only inhibited Smad3 expression. While 4 mg/L of fluoride significantly decreased the expression of p-Smad2/3.SB431542 co-treated with fluoride significantly inhibited protein level ofp-Smad2/3 expression compared with fluoride treatment alone. The inhibitory effect of SB431542 and fluoride was more significant on p-Smad2/3 than that onSmad2/3, which implied that the ALK5 inhibitor significantly impeded the phosphorylation of Smad2/3.

## Discussion

Up to date, role of TGF-β1 signaling on the skeletal fluorosis is was rarely studied. Aberrant change of Runx2 mediated signaling cascade is one of the decisive steps during the pathogenesis of fluorosis. The present study showed that bone mineral density decreased after treated with fluoride for two months, which was similar as previous study [22]. Bone turnover balanced bone mass by regulating osteoblast and osteoclast. Previous study demonstrated bone turnover was activated in rats after treated by fluoride for two months and mainly characterized as active bone resorption mediated by osteoclast [23], which was consistent with our present study. The reduced BMD might mediate by stronger bone turnover, which was consistent with the changes in gene expression of RANKL and Runx2 induced by fluoride treatment. Accompanied with these alterations, as downstream of TGF-β1 signaling, Smad3 showed extremely high expression. However, the decreased expression of ALK5 was consistent with the BMD results. These data implied the association between skeletal fluorosis and TGF-β1. In this study, SB431542 was used to inhibit ALK5, which is one of seven subtypes for TGFβR1 and to provide insight into the mechanism of skeletal fluorosis. Results showed that Runx2 and RANKL expression decreased, along with the Smad3 and AKL5 expression. Accordingly, the value of BMD increased due to inactive bone turnover. These *in vivo* results suggested that TGF-β1 possibly regulated Smad3 pathway in fluoride-induced bone turnover, mainly in bone resorption process.

TGF-β1 is known to regulate majority stages involved in the osteoblast and osteoclast differentiation pathways [24]. In the present study, varied concentrations of NaF were found to induce alterations in cells viability and expression of differentiation related genes and proteins. The present results indicated the dual effect of fluoride on the BMSC viability, which showed that low dose of fluoride stimulated cell viability, but high dose inhibited it. Co-exposure to fluoride and SB431542 impeded cell viability at varying level compared with fluoride treatment alone. Osteoblasts originate from mesenchymal precursors and the osteoblastic differentiation course is regulated by many well-defined genes [25]. Runx2 is one of the key transcription factors for osteoblastic differentiation. In our study, Runx2 expression also was enhanced by low dose of fluoride treatment and inhibited by high dose. Furthermore, ALP activity and OCN protein implied similar dual-action trend as cell viability in BMSC treated by varying doses of fluoride. The ALP activity and OCN are considered as phenotypic markers of osteoblastic differentiation [26,27]. Previous study reported that after rats were treated by fluoride for 15 days, ALP activity was higher with low fluoride treatment compared to control while high fluoride inhibited its activity [28]. Smad3 is an essential factor for TGF-β1 signaling in inhibiting or enhancing Runx2 expression induced by diverse stimuli [29]. Therefore, a decrease of Smad3 expression was observed in fluoride-treated BMSCs, which probably regulated the Runx2 and subsequently influenced osteoblastic viability and differentiation.

Protein analysis showed that SB431542 treatment significantly inhibited phosphorylation of Smad2/3, which implied that ALK5 inhibitor effectively impeded the couple between ALK5 and Smad2/3. Co-treated with fluoride and SB431542 not only reduced the cell viability, ALP activity and OCN expression, but also inhibited expression of Runx2 and ALK5. The low-dose of fluoride and SB431542 treatment greatly reduced the expression of Smad3. However, the reason why high dose of fluoride and SB431542 mildly enhanced Smad3 expression was still unclear. We speculated that less active cells collected in the high-dose fluoride group influenced gene expression analysis. Research indicated that TGF-β1 enhanced ALP activity in MC3T3-E1 cells [30]. The decrease of ALP, OCN and Runx2 *in vitro* closely related with the inhibition of phosphorylation of Smad2/3. To sum up, fluoride influenced bone turnover and BMSC osteoblastic differentiation. Besides, the regulation of fluoride in these processes was influenced by an ALK5 inhibitor. This study suggested fluoride regulated osteoblastic TGF-β1 signaling in bone turnover and BMSCs differentiation via ALK5. Our results provide a theoretical basis for further understanding the mechanism of osteoblastic differentiation induced by fluoride.

## Supporting Information

S1 File All raw data used for drawing result figures. (XLSX)
