# Correction: Fluoride Regulate Osteoblastic Transforming Growth Factor-β1 Signaling by Mediating Recycling of the Type I Receptor ALK5

**DOI:** 10.1371/journal.pone.0176772

**Published:** 2017-04-25

**Authors:** Chen Yang, Yan Wang, Hui Xu

In Fig 2, the y-axis for the graphs Runx2 and RANKL are incorrectly labeled. The axis should be labeled “Rative RNA abundance” Please see the corrected Fig 2 here.

**Fig 2:**
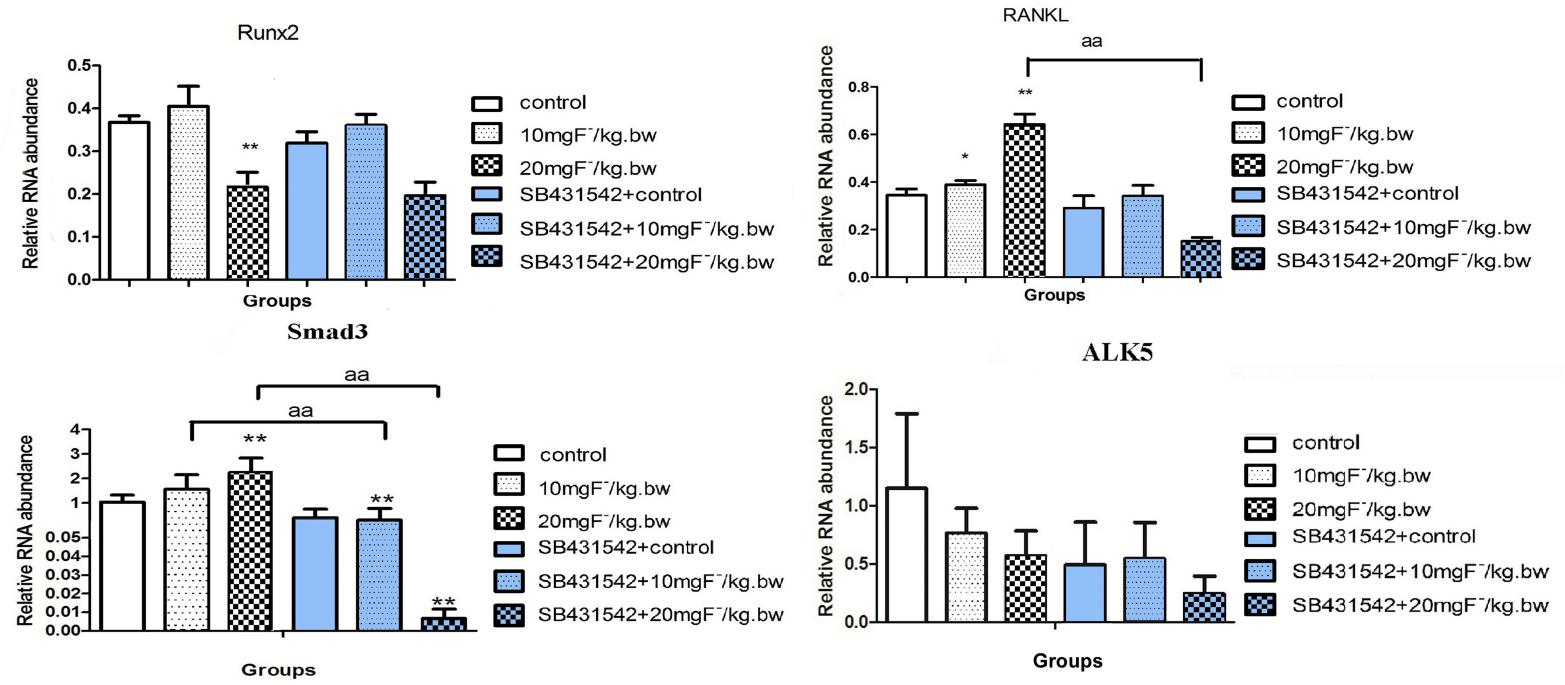
Gene expression of Runx2, RANKL, Smad3 and ALK5 in rats treated by fluoride with or without SB431542. Rats was treated with sodium fluoride by gavage at 10 mgF^-^/kg.bw and 20 mgF^-^/kg.bw for 2 months, and half of rats in each group were injected with an ALK5 inhibitor (SB431542, 2.1 mg/kg.bw). The femurs were collected and extracted mRNA by Trizol reagent. Realtime PCR was used to analyze Runx2, RANKL, Smad3 and ALK5 expression. Results are expressed as mean± SD(n = 3). (**P < 0.01 compare with control group; aa P < 0.01, compare with two groups).

In S1 File, the data for Runx2 and RANKL is incorrect. Please see the corrected S1 File here.

## Supporting information

S1 File All raw data used for drawing result figures. (XLSX)

## References

[1] Yang C, Wang Y, Xu H (2017) Fluoride Regulate Osteoblastic Transforming Growth Factor-β1 Signaling by Mediating Recycling of the Type I Receptor ALK5. PLoS ONE 12(1): e0170674 doi: 10.1371/journal.pone.0170674 28125630 10.1371/journal.pone.0170674 PMC5268439

